# Advances in the field of nanooncology

**DOI:** 10.1186/1741-7015-8-83

**Published:** 2010-12-13

**Authors:** KK Jain

**Affiliations:** 1 Jain PharmaBiotech, Basel, Switzerland

## Abstract

Nanooncology, the application of nanobiotechnology to the management of cancer, is currently the most important chapter of nanomedicine. Nanobiotechnology has refined and extended the limits of molecular diagnosis of cancer, for example, through the use of gold nanoparticles and quantum dots. Nanobiotechnology has also improved the discovery of cancer biomarkers, one such example being the sensitive detection of multiple protein biomarkers by nanobiosensors. Magnetic nanoparticles can capture circulating tumor cells in the bloodstream followed by rapid photoacoustic detection. Nanoparticles enable targeted drug delivery in cancer that increases efficacy and decreases adverse effects through reducing the dosage of anticancer drugs administered. Nanoparticulate anticancer drugs can cross some of the biological barriers and achieve therapeutic concentrations in tumor and spare the surrounding normal tissues from toxic effects. Nanoparticle constructs facilitate the delivery of various forms of energy for noninvasive thermal destruction of surgically inaccessible malignant tumors. Nanoparticle-based optical imaging of tumors as well as contrast agents to enhance detection of tumors by magnetic resonance imaging can be combined with delivery of therapeutic agents for cancer. Monoclonal antibody nanoparticle complexes are under investigation for diagnosis as well as targeted delivery of cancer therapy. Nanoparticle-based chemotherapeutic agents are already on the market, and several are in clinical trials. Personalization of cancer therapies is based on a better understanding of the disease at the molecular level, which is facilitated by nanobiotechnology. Nanobiotechnology will facilitate the combination of diagnostics with therapeutics, which is an important feature of a personalized medicine approach to cancer.

## Introduction

Nanotechnology is the creation and utilization of materials, devices and systems through the control of matter on the nanometer-length scale, that is, at the level of atoms, molecules and supramolecular structures. Given the inherent nanoscale functional components of living cells, it was inevitable that nanotechnology would be applied in biotechnology settings, giving rise to the term *nanobiotechnology*, that is, the application of nanotechnology in the life sciences. Nanobiotechnology is already starting to show the promise of an impact on health care. Nanomedicine is defined as the application of nanobiotechnology to medicine and is based on the use of nanoscale materials and devices for diagnosis and drug delivery as well as for the development of advanced pharmaceuticals referred to as nanopharmaceuticals [1]. Nanobiotechnology is also being applied to refine surgery from microsurgery to nanosurgery. Examples include the construction of nanoscale robots, nanobots, for navigating the human body to detect as well as treat various diseases, and cell surgery using nanodevices and nanolasers. During the past few years, considerable progress has been made in the application of nanobiotechnology in cancer, that is, nanooncology, which is currently the most important chapter of nanomedicine.

**Figure 1:**
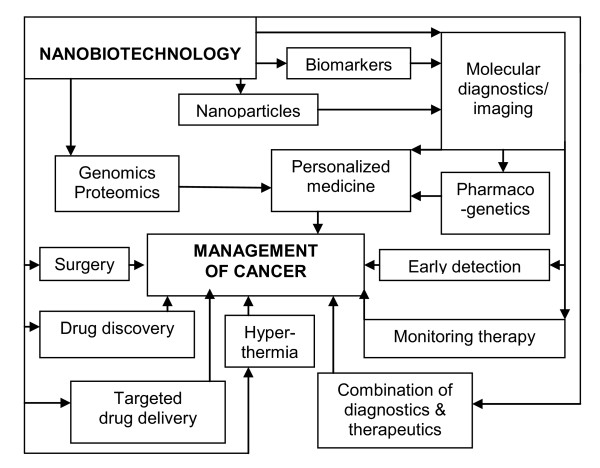
**Role of nanobiotechnology in the management of cancer. **This is a schematic of the role of nanobiotechnology and its interaction with various other technologies and approaches used in the management of cancer. (Reproduced by permission of Jain PharmaBiotech.)

Nanobiotechnology plays an important role in the discovery of biomarkers of cancer. Several drugs in development for cancer are based on nanobiotechnology, and a few of these are already approved. Nanobiotechnology-based devices are in development as aids to cancer surgery. Finally, nanobiotechnology is playing an important role in personalized therapy for cancer. The impact of nanobiotechnology on oncology is shown schematically in Figure 1.

## Role of nanotechnology in cancer diagnostics

Nanobiotechnologies have extended the limits of and refined molecular diagnostics [2]. Nanobiotechnology offers a novel set of tools for the detection of cancer and contributes to early detection of cancer as follows:

1. It can complement existing technologies and make significant contributions to cancer detection, prevention, diagnosis and treatment.

2. It would be extremely useful in the area of biomarker research and provide additional sensitivity in assays with relatively small sample volumes.

3. Examples of applications of nanobiotechnology in cancer diagnostics include quantum dots (QDs) and the use of nanoparticles for tumor imaging.

### Gold nanoparticles for cancer diagnosis

By attaching monoclonal antibodies (mAbs), which can recognize a specific cancer cell, to gold nanoparticles or nanorods the "heating phenomenon" can be used in cancer detection. This acoustic signal gives valuable information about the presence of cancer cells. Gold nanoparticles conjugated to anti-epidermal growth factor receptor (anti-EGFR) mAbs specifically and homogeneously bind to the surface of the cancer cells with 600% greater affinity than to the noncancerous cells. This specific and homogeneous binding is found to give a relatively sharper surface plasma resonance (SPR) absorption band with a red shifted maximum compared to that observed when added to the noncancerous cells [3]. The particles that worked the best in the El-Sayed *et al*. [3] study were 35 nm in size. These results suggest that SPR scattering imaging or SPR absorption spectroscopy generated from antibody conjugated gold nanoparticles may be useful in molecular biosensor techniques for the diagnosis and investigation of oral epithelial cancer cells *in vivo *and *in vitro*. Advantages of using this technique include:

1. Gold nanoparticles are not toxic to human cells. A similar technique with QDs uses semiconductor crystals to mark cancer cells, but the semiconductor material is potentially toxic to the cells and humans.

2. It does not require expensive high-powered microscopes or lasers to view the results. All it takes is a simple, inexpensive microscope and white light.

3. The results are instantaneous. If a cancerous tissue is sprayed with gold nanoparticles containing the antibody, the results can be seen immediately. The scattering is so strong that a single particle can be detected [3].

### Quantum dots for molecular diagnosis of cancer

There is considerable interest in the use of QDs as inorganic fluorophores, owing to the fact that they offer significant advantages over conventionally used fluorescent markers. For example, QDs have fairly broad excitation spectra, from ultraviolet to red, that can be tuned, depending on their size and composition.

The best characteristics of QDs and magnetic iron oxide nanoparticles can be combined to create a single nanoparticle probe that can yield clinically useful images of both tumors and the molecules involved in cancer [4]. In magnetic resonance imaging (MRI) experiments, this combination nanoparticle generated an MRI signal that was over threefold more intense than the same number of iron oxide nanoparticles. These dual-mode nanoparticles were labeled with an antibody that binds to polysialic acid molecules, which are found on the surface of certain lung tumors. These targeted nanoparticles were quickly taken up by cultured tumor cells and were readily visible using fluorescence microscopy.

DNA methylation contributes to carcinogenesis by silencing key tumor suppressor genes. An ultrasensitive and reliable nanotechnology assay, MS-qFRET (fluorescence resonance energy transfer), can detect and quantify DNA methylation [5]. In this method, bisulfite-modified DNA is subjected to polymerase chain reaction (PCR) amplification with primers that would differentiate between methylated and unmethylated DNA. QDs bind to methylated DNA strands, which then light up and are identifiable via a spectrophotometer. A MS-qFRET test of blood samples can be used for noninvasive cancer screening. It can also be used to determine whether a cancer treatment is effective and thus enable personalized chemotherapy.

### Nanotechnology for detection of cancer biomarkers

Any specific molecular alteration of a cell on the DNA, RNA, metabolite or protein level may be referred to as a molecular biomarker. From a practical point of view, the biomarker would specifically and sensitively reflect a disease state and could be used for diagnosis as well as for disease monitoring during and following therapy [6]. Currently available molecular diagnostic technologies have been used to detect biomarkers of various diseases such as cancer. Nanotechnology has further refined the detection of biomarkers. The physicochemical characteristics and high surface areas of nanoparticles make them ideal candidates for developing platforms for harvesting biomarkers. Some biomarkers also form the basis of innovative molecular diagnostic tests.

A magnetic nanosensor technology is up to 1,000 times more sensitive than any technology now in clinical use, can detect biomarker proteins over a range of concentrations three times broader than any existing method and is accurate regardless of which bodily fluid is being analyzed [7]. The nanosensor chip also can search for up to 64 different proteins simultaneously and has been shown to be effective in early detection of tumors in mice, suggesting that it may open the door to significantly earlier detection of even the most elusive cancers in humans. The magnetic nanosensor can successfully detect cancers in mice when levels of cancer-associated proteins are still well below concentrations detectable using the current standard method, the enzyme-linked immunosorbent assay. The sensor also can be used to detect biomarkers of diseases other than cancer.

### Investigating the potential for capturing circulating tumor cells

A method has been described for magnetically capturing circulating tumor cells in the bloodstream of mice followed by rapid photoacoustic detection [8]. Magnetic nanoparticles, which were functionalized to target a receptor commonly found in breast cancer cells, bound and captured circulating tumor cells under a magnet. To improve detection sensitivity and specificity, gold-plated carbon nanotubes conjugated with folic acid were used as a second contrast agent for photoacoustic imaging. Through integrating *in vivo *multiplex targeting, magnetic enrichment, signal amplification and multicolor recognition, this approach enables circulating tumor cells to be concentrated from a large volume of blood in the vessels of tumor-bearing mice and has potential applications for the early diagnosis of cancer and the prevention of metastasis in humans.

### Imaging applications of nanobiotechnology in cancer

Highly lymphotropic superparamagnetic iron oxide nanoparticles (SPIONs), measuring 2 to 3 nm on average (Combidex, Advanced Magnetics, Cambridge, Mass, USA), gain access to lymph nodes by means of interstitial lymphatic fluid transport. SPIONs have been used in conjunction with high-resolution MRI to reveal small and otherwise undetectable lymph node metastases. In patients with prostate cancer who undergo surgical lymph node resection or biopsy, MRI with lymphotropic SPIONs can identify all patients with nodal metastases. This is important for the management of cancer, but is not possible with conventional MRI alone. Occult lymph node metastases in patients with prostate cancer have been identified by this technique prior to salvage radiation therapy [9].

QDs are attractive as optical imaging agents owing to their high brightness and photo- and biostability. Bioluminescence resonance energy transfer QDs can improve the signal-to-background ratio for real-time imaging largely by suppressing background signal [10].

### Nanotechnology-based drugs for cancer

Approximately 150 drugs in development for cancer are based on nanotechnology. Some of these are already approved, as shown in Table 1, and the rest are in various stages of development.

**Table 1:** Approved anticancer drugs using nanocarriers^a^

Trade name/compound	Manufacturer	Nanocarrier
Abraxane/paclitaxel	Abraxis Biosciences	Albumin-bound paclitaxel
Bexxar/anti-CD20 conjugated to iodine-131	Corixa/GlaxoSmithKline	Radioimmunoconjugate
DaunoXome/daunorubicin	Diatos (available in France)	Liposome
Doxil/Caelyx/doxorubicin	Ortho Biotech	Liposome
Myoset/doxorubicin	Cephalon (available in Europe)	Nonpegylated liposome
Oncaspar/PEG-L-asparaginase	Enzon	Polymer-protein conjugate
Ontak/IL-2 fused to diphtheria toxin	Eisai Inc	Immunotoxic fusion protein
SMANCS/zinostatin	Yamanouchi Pharma	Polymer-protein conjugate
Zevalin/anti-CD20 conjugated to yttrium-90	Cell Therapeutics Inc.	Radioimmunoconjugate
Zoladex/goserelin acetate	AstraZeneca	Polymer rods

^a^PEG, polyethylene glycol; IL-2, interleukin-2; SMANCS, styrene maleic anhydride neocarzinostatin. Reproduced by permission ^© ^Jain PharmaBiotech.

### Current clinical trials using nanobiotechnology for cancer

Clinical trial phases of anticancer drugs based on nanobiotechnology are shown in Table 2. Not all of the results of the clinical trials have been published yet, but those that are available are described in the following text.

**Table 2:** Clinical trials of anticancer drugs using nanocarriers^a^

Compound	Nanocarrier	Trial stage
CPX-1 irinotecan	Liposome	Phase I [11]
LE-SN38 irinotecan	Liposome	Phase II colorectal cancer
MCC465 doxorubicin	mAb-liposome	Phase I [12]
NC-6004 cisplatin	Micelle	Phase II [13]
NK105 paclitaxel	Micelle	Phase II
NK911 doxorubicin	Micelle	Phase I
PK1 doxorubicin	HPMA copolymer	Phase II/III [14]
SP1049C doxorubicin	Micelle	Phase III [15]

^a^mAb, monoclonal antibody; HPMA, N-(2-hydroxypropyl)methacrylamide. Reproduced by permission ^© ^Jain PharmaBiotech. References to clinical trials are given in section "Current clinical trials using nanobiotechnology for cancer" of the main article.

### CPX-1

CPX-1 is a novel liposome-encapsulated formulation of irinotecan and floxuridine designed to prolong *in vitro *optimized synergistic molar ratios of both drugs following infusion. An open-label, single-arm, dose-escalating phase I study was conducted to determine the maximum tolerated dose and pharmacokinetics of CPX-1 in patients with advanced solid tumors [11]. The results showed that CPX-1 was well tolerated, and antitumor activity was shown in patients with advanced solid tumors.

### MCC-465

MCC-465 is an immunoliposome-encapsulated doxorubicin (DOX). The liposome is tagged with polyethylene glycol (PEG) and the F(ab")_2 _fragment of human mAb GAH (goat anti-human), which positively reacts to >90% of cancerous stomach tissues but negatively to all normal tissues. In preclinical studies, MCC-465 showed superior cytotoxic activity against several human stomach cancer cells compared with DOX or DOX-incorporated PEG liposomes. A phase I clinical trial was conducted to define the maximum tolerated dose, dose-limiting toxicity, recommended phase II dose and pharmacokinetics of MCC-465 [12]. The results showed that MCC-465 was well tolerated, and the recommended dose for a phase II study was considered to be 32.5 mg/m^2 ^in an equivalent amount of DOX. A phase II trial was recommended, but further information is currently not available.

### NC-6004

Polymeric micelles are expected to increase the accumulation of drugs in tumor tissues utilizing the EPR (enhanced permeability and retention) effect and to incorporate various kinds of drugs into the inner core by chemical conjugation or physical entrapment with relatively high stability. The size of the micelles can be controlled within the diameter range of 20-100 nm to ensure that the micelles do not pass through normal vessel walls; therefore, a reduced incidence of the side effects of the drugs may be expected as a result of the decreased volume of distribution. There are several anticancer agent-incorporated micelle carrier systems under clinical evaluation. Phase I studies of a CDDP (cisplatin) -incorporated micelle, NC-6004, and a phase II study of a PTX (Paclitaxel) -incorporated micelle, NK105 for stomach cancer, are in progress [13].

### PK1 doxorubicin

Phase I studies of polymer doxorubicin (PK1) showed signs of activity coupled with fivefold decreased anthracycline toxicity in chemotherapy-refractory patients. Phase II studies were conducted using a similar material in patients with breast cancer, non-small cell lung cancer and colorectal cancer [14]. The results showed efficacy with limited side effects, supporting the concept that polymer-bound drugs can improved anticancer activity. PK1 is now in phase III clinical trials.

### SP1049C doxorubicin

The antitumor activity of SP1049C, a novel P-glycoprotein targeting micellar formulation of doxorubicin consisting of DOX and two nonionic block copolymers, has been evaluated in patients with advanced adenocarcinomas of the esophagus and gastroesophageal junction [15]. SP1049C had a notable single-agent activity in patients with adenocarcinomas of the esophagus and gastroesophageal junction, as well as an acceptable safety profile. These results, in addition to the results of preclinical studies, demonstrate superior antitumor activity of SP1049C compared with DOX in a standard formulation. Phase III clinical trials are now in progress.

### Role of nanobiotechnology in therapeutic delivery for cancer

Nanoparticle formulations help to overcome the issue of drug solubility, which is an essential factor for drug effectiveness. Another advantage is facilitation of drug delivery across various barriers, the most important of which is the blood-brain barrier, which limits access to brain tumors. Other major advantages include targeted drug delivery, use of lower doses with reduced toxicity and facilitation of a combination of diagnostics with therapeutics for cancer. Several nanobiotechnologies, mostly based on nanoparticles, have been used to facilitate drug delivery in cancer, and a classification is shown in Table 3.

**Table 3:** Classification of nanobiotechnology approaches to drug delivery in cancer^a^

Approach	Examples
Nanoparticles	Nanoparticle formulations of anticancer drugs, for example, paclitaxel
Exosomes for cancer drug delivery	Dexosomes (exosomes produced by dendritic cells) as cancer vaccines
Nanoencapsulation and enclosure of anticancer drugs	Enclosing drugs in lipid nanocapsules
	Encapsulating drugs in hydrogel nanoparticles
	Micelles for drug delivery in cancer
Targeted delivery of anticancer therapy	Targeted drug delivery with nanoparticles
	Pegylated nanoliposomal formulation
	Folate-linked nanoparticles
	Carbon magnetic nanoparticles for targeted drug delivery
	Nanoparticle-aptamer bioconjugates
	Nanodroplets for site-specific cancer treatment
	Lipid-based nanocarriers
	Targeted antiangiogenic therapy using nanoparticles
	Nanoparticles for delivery of drugs to brain tumors
Combination of nanoparticles with radiotherapy	Combination with boron neutron capture therapy
	Nanoengineered silicon for brachytherapy
Combination with physical modalities of cancer therapy	Combination with laser ablation of tumors
	Combination with photodynamic therapy
	Combination with thermal ablation
	Combination with ultrasound
Nanoparticle-mediated gene therapy	*p53 *gene therapy of cancer
	Immunolipoplex for delivery of *p53 *gene
	Intravenous delivery of *FUS1 *gene
Combination of diagnostics and therapeutics	Nanoshells as adjuncts to thermal tumor ablation
	Perfluorocarbon nanoparticles
	Nanocomposite devices

^a^From Jain KK: *The Handbook of Nanomedicine*, Totowa, NJ: Humana/Springer; 2008 (reproduced by permission). ^© ^Jain PharmaBiotech.

### Nanoparticle formulations of anticancer drugs

An example of nanoparticle formulations of anticancer drugs is paclitaxel, which is widely used to treat multiple types of solid tumors. The commercially available paclitaxel formulation uses Cremophor/ethanol (C/E) as the solubilizer. A study evaluated the effects of nanoparticle formulation of paclitaxel on its tissue distribution in experimental animals [16]. The nanoparticle and C/E formulations showed significant differences in paclitaxel disposition: The nanoparticles yielded 40% smaller area under the blood concentration-time curve and faster blood clearance of total paclitaxel concentrations (sum of free, protein-bound and nanoparticle-entrapped drug). The tissue specificity of the two formulations was different. The nanoparticles showed longer retention and higher accumulation in organs and tissues. Solid tumors have unique features, such as leaky tumor blood vessels and defective lymphatic drainage, that promote the delivery and retention of particles, a phenomenon recognized as the enhanced permeability and retention effect. Nanoformulation can more easily enter and accumulate within tumor cells. This means that higher doses of the drug can be delivered, increasing its anticancer effects while decreasing the side effects associated with systematic chemotherapy. However, there are many variable factors, such as clearance of nanoparticles in the circulation by the kidneys and trapping by reticuloendothelial cells, that affect the amount of anticancer nanoparticles retained in the tumor. One way to overcome some of these variables is targeted drug delivery.

Gold nanoparticles (2 nm) have been covalently functionalized with paclitaxel [17]. The organic shell of hybrid nanoparticles contains 67% by weight of paclitaxel, which corresponds to ~70 molecules of the drug per 1 nanoparticle. This approach provides an opportunity to prepare hybrid particles with a well-defined amount of drug and offers a new alternative for the design of nanosize drug-delivery systems. Follow-up studies will determine the potency of the paclitaxel-loaded nanoparticles. Since each ball is loaded with a uniform number of drug molecules, it will be relatively easy to compare the effectiveness of the nanoparticles with the effectiveness of generally administered paclitaxel. This technique could help to deliver more of the drug directly to the cancer cells and reduce the side effects of chemotherapy. The aim is to improve the effectiveness of the drug by increasing its ability to stay bound to microtubules within the cell.

The best known of the approved drugs is nanoparticle albumin-bound paclitaxel (Abraxis Biosciences'/Celgene, Summit, NJ, USA) Abraxane, which is based on proprietary Protosphere nanoparticle technology, also referred to as nanoparticle albumin-bound or nab™ technology. This technology integrates biocompatible proteins with drugs to create the nanoparticle form of the drug having a size of about 100-200 nm. SPARC (secreted protein acidic and rich in cysteine), a protein overexpressed and secreted by cancer cells, binds albumin to concentrate albumin-bound cytotoxic drugs at the tumor. The solvent Cremophor-EL, used previously in formulations of paclitaxel, causes severe hypersensitivity reactions. Because Abraxane is solvent-free, solvent-related toxicities are eliminated and administration can occur more rapidly. Abraxane also has a different toxicity profile than solvent-based paclitaxel, including a lower rate of severe neutropenia. A pivotal randomized, controlled phase III clinical trial compared the safety and efficacy of 260 mg/m^2 ^of Abraxane to 175 mg/m^2 ^of Taxol administered every 3 weeks in patients with metastatic breast cancer [18]. Abraxane was found to be superior to Taxol with regard to lesion response rate as well as tumor progression rate. It was approved for the treatment of metastatic breast cancer and is being evaluated in non-small cell lung cancer, ovarian cancer, melanoma and cervical cancer.

## Targeted drug delivery using nanobiotechnology

### Principles of targeted drug delivery

The basic principle of targeted drug delivery in cancer using nanoparticles injected intravenously into the blood circulation is that the nanoparticles should recognize the receptors on the cancer cell, anchor themselves to it and diffuse inside the cell. Once inside, the nanoparticle disintegrates, causing a nearly instantaneous release of the drug precisely where it is needed. Nanoparticles can be chemically programmed to have an affinity for the cell wall of tumors or attached to mAb ligands that bind specifically to receptors on tumor cells. To be effective, the particles must evade the body's immune system, penetrate into the cancer cells and discharge the drugs before being recognized by the cancer cells.

Nanosystems are emerging that may be very useful for tumor-targeted drug delivery. Novel nanoparticles are preprogrammed to alter their structure and properties during the drug delivery process to make them most effective for delivery [19]. This alteration can be achieved through the incorporation of molecular sensors that are able to respond to physical or biological stimuli, including changes in pH, redox potential or enzymes.

Nanoparticles are suitable for two specific tasks required for targeted drug delivery to pathological sites in the body: (1) passive enhanced permeability and retention based on the longevity of the pharmaceutical carrier in the blood, leading to its accumulation in pathological sites with compromised vasculature, and (2) active targeting based on the attachment of specific ligands to the surface of pharmaceutical carriers to recognize and bind pathological cells [20]. Differentially expressed molecules at receptors can be used as docking sites to concentrate drug conjugates and nanoparticles at tumor sites [21].

### Targeted delivery of biological therapies for cancer

Physical forces (for example, electric or magnetic fields, ultrasound, hyperthermia or light) may contribute to focusing and triggered activation of nanosystems. Biological drugs delivered with programmed nanosystems also include plasmid DNA, small interfering RNA (siRNA) and other therapeutic nucleic acids.

A tumor-targeted nanodrug consisting of SPIONs (MN-EPPT-siBIRC5), which is designed to specifically carry siRNA to human breast tumors, binds the tumor-specific antigen uMUC-1 found on over 90% of human breast adenocarcinomas, translating into a significant decrease in tumor growth rate [22]. Following intravenous injection, the nanodrug demonstrates a preferential uptake by tumor, which can be visualized by MRI and near-infrared (NIR) optical imaging. This approach enables simultaneous targeted delivery of siRNA to tumors and the imaging of the delivery process.

### Cyclosert system for targeted delivery of anticancer therapeutics

Cyclosert™ (Calando Pharmaceuticals, Pasadena, CA, USA) is the first nanoparticle drug transport platform to be designed *de novo *and synthesized specifically to overcome limitations in existing technologies used for the systemic transport of therapeutics to targeted sites within the body. Based on small cyclic repeating molecules of glucose called cyclodextrins, Cyclosert promotes the ability of cytotoxic drugs to inhibit the growth of human cancer cells while reducing toxicity and remaining nonimmunogenic cells at therapeutic doses. In particular, the system is designed to reduce the toxicity of the drugs until they actually reach the targeted tumor cells, where the active drug is released in a controlled fashion. Animal studies have shown that the Cyclosert system can safely deliver tubulysin A, a potent but highly toxic antitumor agent. *In vitro *studies have shown the tubulysin-Cyclosert conjugate to be effective against multiple human cancer cell lines. The conjugate is stable and 100 times more water-soluble than the free drug. Calando (Pasadena, CA, USA) is developing CALAA01, a siRNA, for anticancer therapy using Cyclosert as a delivery system.

IT-101 (Calando) is a *de novo *designed experimental therapeutic composed of linear, cyclodextrin (CD)-containing polymer conjugates of camptothecin (CPT) that assemble into 40-nm-diameter nanoparticles via polymer-polymer interactions that involve inclusion complex formation between the CPT and the CD. Particle size, near-neutral surface charge and CPT release rate were specifically designed into IT-101. The Cyclosert platform forms nanoscale constructs with hydrodynamic diameters between 30 and 60 nm. This makes Cyclosert-based drugs ideal for effective delivery to solid tumors. Preclinical animal studies have shown extended circulation times, tumor accumulation, slow release of the CPT and anticancer efficacy that directly correlate to the properties of the nanoparticle. Release of CPT can disassemble the nanoparticle into individual polymer chains ~10 nm in size that are capable of renal clearance. IT-101 has been evaluated in patients with relapsed or refractory cancer following two cycles of therapy by intravenous infusion. Interim analysis has shown that IT-101 is well tolerated and that pancytopenia is the dose-limiting toxicity [23]. Pharmacokinetic data were favorable and consistent with results from preclinical animal studies. In the patients studied, IT-101 showed longer half-life, lower clearance and lower volume of distribution than that seen in patients treated with other camptothecin-based drugs. IT-101 is in phase II clinical trials.

### Ultrasonic tumor imaging and targeted chemotherapy by nanobubbles

Drug delivery in polymeric micelles combined with tumor irradiation by ultrasound results in effective drug targeting, but this technique requires prior tumor imaging. A new targeted drug delivery method for doxorubicin used ultrasound to image tumors while also releasing the drug from nanobubbles into the tumor implants in mice [24]. At physiologic temperatures, nanodroplets converted into nanobubbles. Doxorubicin was localized in the nanobubble walls formed by the block copolymer. Upon intravenous injection into mice, DOX-loaded micelles and nanobubbles extravasated selectively into the tumor interstitium, where the nanobubbles coalesced to produce microbubbles. When exposed to ultrasound, the bubbles generated echoes, which made it possible to image the tumor. The sound energy from the ultrasound popped the bubbles, releasing DOX, which enhanced intracellular uptake by tumor cells *in vitro *to a statistically significant extent compared to that observed with nanobubbles that were not exposed to ultrasound.

In summary, multifunctional nanoparticles that are tumor-targeted drug carriers, long-lasting ultrasound contrast agents and enhancers of ultrasound-mediated drug delivery have been developed and deserve further exploration as cancer therapeutics.

### Nanoparticle-based thermal ablation of cancer

Several forms of energy have been used for the destruction of tumor cells that cannot be reached for conventional surgical excision. Thermal ablation therapy is the most promising of these methods but is limited by incomplete tumor destruction and damage to adjacent normal tissues. Current radiofrequency ablation techniques require invasive needle placement and are limited by accuracy of targeting. Use of nanoparticles has refined noninvasive thermal ablation of tumors, and several nanomaterials have been used for this purpose. These include gold nanomaterials, iron nanoparticles, magnetic nanoparticles, carbon nanotubes and affisomes. Heating of the particles can be induced by magnets, lasers, ultrasound, photodynamic therapy and low-power X-rays [1].

In addition to delivering heat, hyperthermia offers additional treatment options by enhancing the effects of chemoradiation treatments. A lower radiation dose is required to kill the same fraction of cells when they are also subjected to hyperthermia. This is because hypoxic cells are resistant to radiation, but heat destroys hypoxic cells as efficiently as normal cells [1].

### Gold nanomaterials for thermal ablation

Noninvasive radiowave thermal ablation of cancer cells is feasible when facilitated by gold nanoparticles [25]. CYT-6091, a pegylated colloidal gold nanoparticle containing tumor necrosis factor-α bound to its surface, has been extensively investigated as an adjuvant and has been shown to enhance thermal therapies [26]. This technique could be adapted to *in vivo *use.

Photothermal therapy can be used to heat gold nanoparticles to destroy the tumor, while another option would be to include gold particles in capsules filled with cancer medication. The capsule attaches to the cancer cell and is heated, and the medicine is released locally. Plasmon-resonant gold nanorods, which have large absorption cross sections at NIR frequencies, are very effective as multifunctional agents for image-guided cancer therapies based on localized hyperthermia. Nanorods coated with cetyltrimethylammonium bromide (a cationic surfactant used in nanorod synthesis) are internalized quickly into cancer cells by a nonspecific uptake pathway, whereas the removal of cetyltrimethylammonium bromide from nanorods functionalized with folate results in their accumulation on the cell surface over the same time interval [27]. Thus the nanorods render the tumor cells highly susceptible to photothermal damage when irradiated at the nanorods' longitudinal plasmon resonance.

Gold nanoshell-based, targeted, multimodal contrast agents in NIR are fabricated and utilized as a diagnostic and therapeutic probe for MRI, fluorescence optical imaging and photothermal cancer therapy of breast carcinoma cells *in vitro *[28]. This may enable diagnosis as well as treatment of cancer during one hospital visit.

### Magnetic nanoparticles for thermal ablation of tumors

Magnetic thermal ablation has been examined under *in vivo *animal conditions. Magnetic nanoparticles are promising tools for minimally invasive elimination of small tumors in the breast using magnetically induced heating. The approach complies with the increasing demand for breast-conserving therapies and has the advantage of offering a selective and refined tuning of the degree of energy deposition, allowing an adequate temperature control at the target [29].

Anti-human epidermal growth factor receptor 2 (anti-HER2) antibody can induce antitumor responses and can be used in delivering drugs to HER2-overexpressing cancer. Anti-HER2 immunoliposomes containing magnetite nanoparticles, which act as tumor-targeting vehicles, have been used to combine anti-HER2 antibody therapy with hyperthermia [30]. When introduced into SKBr3 breast cancer cells *in vitro*, 60% of magnetite nanoparticles incorporated into the SKBr3 cells. The cells were then heated at 42.5°C under an alternating magnetic field, resulting in strong cytotoxic effects. These results suggest that this novel therapeutic tool is applicable to the treatment of HER2-overexpressing cancer.

Magnetorelaxometry can also be used to monitor the accumulation of magnetic nanoparticles before cancer therapy, with magnetic heating being an important precondition for treatment success [31]. Although nanoparticle-mediated thermal therapy is a promising treatment of cancers, challenges posed by this form of hyperthermia include the nontarget biodistribution of nanoparticles in the reticuloendothelial system when administered systemically, the inability to visualize or quantify the global concentration and spatial distribution of these particles within tumors, the lack of standardized thermal modeling as well as algorithms for determining dose, and the concerns regarding their biocompatibility [32].

### Laser-induced thermal destruction of cancer using nanoparticles

Single-walled carbon nanotubes (SWCNTs) show strong optical absorbance 700- to 1,100-nm NIR laser impulses. SWCNTs emit heat when they absorb energy from NIR light. Tissue is relatively transparent to NIR, which suggests that targeting SWCNTs to tumor cells, followed by noninvasive exposure to NIR light, will ablate tumors within the range of NIR. One study has demonstrated the specific binding of mAb-coupled SWCNTs to tumor cells *in vitro*, followed by their highly specific ablation with NIR light [33]. Only the specifically targeted cells were killed after exposure to NIR light. Selective cancer cell destruction can be achieved by functionalization of SWCNTs with a folate moiety, selective internalization of carbon nanotubes inside cells labeled with folate receptor tumor biomarkers, and NIR-triggered cell death, without harming receptor-free normal cells.

### Photodynamic therapy of cancer using nanoparticles

Poly(lactic-co-glycolic acid) nanoparticles can encapsulate the photosensitizer mesotetraphenylporpholactol and are not phototoxic upon systemic administration, but upon cellular internalization the photosensitizer is released from the nanoparticle and becomes highly phototoxic. *In vivo *experiments have shown complete eradication of cancers by photodynamic therapy (PDT) in mouse models.

A nanocarrier consisting of polymeric micelles of diacylphospholipid-poly(ethylene glycol) (PE-PEG) coloaded with the photosensitizer drug 2-[1-hexyloxyethyl]-2-devinyl pyropheophorbide and magnetic Fe_3_O_4 _nanoparticles have been used for guided drug delivery, together with light-activated PDT, for cancer [34]. The magnetophoretic control on the cellular uptake provides enhanced imaging and phototoxicity. These multifunctional nanocarriers demonstrate the exciting prospect offered by nanochemistry for targeting PDT.

In a novel nanoformulation for PDT of cancer, the photosensitizer molecules are covalently incorporated into organically modified silica nanoparticles [35]. These incorporated photosensitizer molecules retain their spectroscopic and functional properties and can robustly generate cytotoxic singlet oxygen molecules upon photoirradiation. The advantage offered by this covalently linked nanofabrication is that the drug is not released during systemic circulation, which is often a problem with physical encapsulation. These nanoparticles are also avidly taken up by tumor cells and demonstrate phototoxic action, thereby improving the diagnosis as well as PDT of cancer, the efficacy of which remains controversial for some types of cancer.

### Targeted delivery of thermosensitive affibody-conjugated liposomes for cancer

Thermosensitive liposomes have been used as vehicles for the delivery and release of drugs to tumors. To improve the targeting efficacy for breast cancer treatment, a HER2-specific affibody molecule was conjugated to the surface of thermosensitive small unilamellar liposomes measuring 80-100 nm, referred to as "Affisomes," to study the effects of this modification on physical characteristics and the stability of the resulting preparation [36]. Affisomes released calcein, a water-soluble fluorescent probe, in a temperature-dependent manner, with optimal leakage (90%-100%) at 410°C. Affisomes are therefore promising candidates for targeted thermotherapy of breast cancer.

### Role of nanotechnology in personalized therapy of cancer

Personalized medicine simply means the prescription of specific therapeutics best suited for an individual. The scope of personalized medicine is much broader than that indicated by the term *genomic medicine*. Personalized management is usually based on pharmacogenetic, pharmacogenomic, pharmacoproteomic and pharmacometabolic information, but other individual variations in patients and environmental factors are also taken into consideration [37]. In cancer cases, the variation in behavior of cancer of the same histological type from one patient to another is also taken into consideration. Personalization of cancer therapies is based on a better understanding of the disease at the molecular level, and nanotechnology will play an important role in this area [38]. With so many nanotechnologies available for drug delivery, it is recommended that computational mathematical tools be used to predict which nanovectors, surface modifications, therapeutic agents and penetration enhancers to use for a multistage drug-delivery strategy that would enable efficient localized delivery of chemotherapeutic drugs and lead to significant improvements in therapy efficacy as well as reduced systemic toxicity [39]. Such an approach can be optimized for personalized oncology.

### Combination of diagnostics with therapeutics

The refinement of molecular diagnostics and the integration of diagnosis with therapy are important features of personalized medicine. As described in the sections on diagnosis and targeted drug delivery, use of the same nanoparticle for diagnosis as well as therapy enables integration of two important facets of cancer management. Dendrimers can be used as advanced contrast agents for imaging techniques such as MRI and can be targeted specifically to cancer cells. Dendrimers can also be used to deliver a variety of cancer therapies to improve their safety and efficacy. For example, applications of dendrimers in photodynamic therapy, boron neutron capture therapy and gene therapy for cancer are being investigated [40].

A biocompatible, multimodal iron oxide nanoparticle has been synthesized for targeted cancer therapy and for optical imaging as well as MRI. A modified solvent diffusion method is used for the coencapsulation of both an anticancer drug and NIR dyes [41]. The resulting folate-derived nanoparticles combining diagnostic and therapeutic properties can be used for imaging as well as targeted killing of folate-expressing cancer cells.

## Concluding remarks

The rationale for using nanobiotechnology in oncology is that nanoparticles have optical, magnetic or structural properties that are not available from larger molecules or bulk solids. When linked with tumor-targeting ligands such as mAbs, peptides or small molecules, these nanoparticles can be used to target tumor antigens (biomarkers) as well as tumor vasculatures with high affinity and specificity [42]. Nanoparticles measuring 5-100 nm in diameter have sufficiently large surface areas and functional groups for conjugating to multiple diagnostic and therapeutic anticancer agents. Recent advances have led to bioaffinity nanoparticle probes for molecular and cellular imaging, targeted nanoparticle drugs for cancer therapy and integrated nanodevices for early cancer detection and screening. Nanobiotechnology has contributed significantly to the diagnosis and therapy of cancer and, by enabling a combination of these, will facilitate the development of personalized management of cancer. Nanobiotechnology has facilitated the discovery of biomarkers that can be used to diagnose and treat cancer based on the molecular profiles of individual patients. Nanoparticles enable targeted delivery of cancer therapeutics and increase efficacy as well as reduce adverse effects.

Many challenges still remain to be resolved prior to widespread use of nanobiotechnology in clinical oncology. There is some concern about the toxicity of nanoparticles, and extensive investigations are in progress to resolve this issue. There is no consensus on the real risks of nanomaterials. Risk evaluation presents challenges because of a lack of data, the complexity of nanomaterials, measurement difficulties and undeveloped hazard assessment frameworks. This topic is discussed in detail in a chapter of a special report on nanobiotechnology [43]. Detailed discussion is beyond the scope of this article, but some of the conclusions of this review are listed below:

1. The risk of nanoparticles depends on their type; some are toxic, whereas others have negligible toxicity and some even have a tissue-protective effect.

2. Measures are available to reduce the toxicity of nanoparticles.

3. The use of biodegradable polymer nanoparticles is suitable for drug delivery as there is no significant toxicity.

Nanooncology has a promising future, and further advances are anticipated in the next 5 years. It is feasible to use molecular tools to design a miniature robotic device, a nanobot, that can be introduced in the body to locate and identify cancer cells and finally destroy them. The device would have a biosensor to identify cancer cells and a supply of anticancer substance that could be released on encountering cancer cells. A small computer could be incorporated to program and integrate the combination of diagnosis and therapy and provide the possibility of monitoring the *in vivo *activities by an external device. Since there is no universal anticancer agent, the computer program could match the type of cancer to the most appropriate agent. Such a device could be implanted as a prophylactic measure in people who do not show any obvious manifestations of cancer. It would circulate freely and could detect and treat cancer at the earliest stage. Such a device could be reprogrammed through removal control and enable a change of strategy if the lesion encountered is other than cancer [44].

## Abbreviations

ELISA: enzyme-linked immunosorbent assay; mAb: monoclonal antibody; MRI: magnetic resonance imaging; MS: mass spectrometry; NIR: near-infrared; PCR: polymerase chain reaction; PDT: photodynamic therapy; QD: quantum dots; SPR: surface plasma resonance; SWCNT: single wall carbon nanotube.

## Competing interests

The authors declare that they have no competing interests.

## Author's information

KKJ, a retired professor of neurosurgery, has been involved in biotechnology for several years with a focus on translation into clinical medicine and integration of technologies to develop personalized medicine. KKJ is the author of 425 publications, including 17 books and 49 special reports on biopharmaceutical topics, including nanobiotechnology. His publications include articles in several scientific journal as well as book chapters on nanobiotechnology and nanomedicine. A book on the role of nanobiotechnology in molecular diagnostics was published in 2006 and *Handbook of Nanomedicine *in 2008 by Humana/Springer Biosciences. Editorial board membership includes the journals *Nanomedicine*, *Technology in Cancer Research & Treatment*, and *Journal of Nanoneuroscience*. He is an invited lecturer on nanobiotechnology as well as a member of review/advisory panels for research grants by various government agencies in the United States (U.S. Army), Canada, the European Union, the Netherlands and Singapore.

## Pre-publication history

The pre-publication history for this paper can be accessed here:

http://www.biomedcentral.com/1741-7015/8/83/prepub
